# Population Pharmacokinetic Model Development of Tacrolimus in Pediatric and Young Adult Patients Undergoing Hematopoietic Cell Transplantation

**DOI:** 10.3389/fphar.2021.750672

**Published:** 2021-12-07

**Authors:** Jordan T. Brooks, Ron J. Keizer, Janel R. Long-Boyle, Sandhya Kharbanda, Christopher C. Dvorak, Brian D. Friend

**Affiliations:** ^1^ Department of Clinical Pharmacy, School of Pharmacy, University of California, San Francisco, San Francisco, CA, United States; ^2^ Insight RX, Inc, San Francisco, CA, United States; ^3^ Department of Pediatrics, University of California, San Francisco, San Francisco, CA, United States; ^4^ Department of Pediatrics, Center for Cell and Gene Therapy, Baylor College of Medicine, Houston, TX, United States

**Keywords:** population pharmacokinetic model, pediatrics, tacrolimus, hematopoietic stem cell transplantation, therapeutic drug monitoring

## Abstract

**Background:** With a notably narrow therapeutic window and wide intra- and interindividual pharmacokinetic (PK) variability, initial weight-based dosing along with routine therapeutic drug monitoring of tacrolimus are employed to optimize its clinical utilization. Both supratherapeutic and subtherapeutic tacrolimus concentrations can result in poor outcomes, thus tacrolimus PK variability is particularly important to consider in the pediatric population given the differences in absorption, distribution, metabolism, and excretion among children of various sizes and at different stages of development. The primary goals of the current study were to develop a population PK (PopPK) model for tacrolimus IV continuous infusion in the pediatric and young adult hematopoietic cell transplant (HCT) population and implement the PopPK model in a clinically available Bayesian forecasting tool.

**Methods:** A retrospective chart review was conducted of 111 pediatric and young adult patients who received IV tacrolimus by continuous infusion early in the post-transplant period during HCT from February 2016 to July 2020 at our institution. PopPK model building was performed in NONMEM. The PopPK model building process included identifying structural and random effects models that best fit the data and then identifying which patient-specific covariates (if any) further improved model fit.

**Results:** A total of 1,648 tacrolimus plasma steady-state trough concentrations were included in the PopPK modeling process. A 2-compartment structural model best fit the data. Allometrically-scaled weight was a covariate that improved estimation of both clearance and volume of distribution. Overall, model predictions only showed moderate bias, with minor under-prediction at lower concentrations and minor over-prediction at higher predicted concentrations. The model was implemented in a Bayesian dosing tool and made available at the point-of-care.

**Discussion:** Novel therapeutic drug monitoring strategies for tacrolimus within the pediatric and young adult HCT population are necessary to reduce toxicity and improve efficacy in clinical practice. The model developed presents clinical utility in optimizing the use of tacrolimus by enabling model-guided, individualized dosing of IV, continuous tacrolimus *via* a Bayesian forecasting platform.

## Introduction

Acute graft-versus-host disease (aGVHD) is a major cause of morbidity and mortality in patients who have undergone allogeneic hematopoietic cell transplantation (HCT). In T cell-replete transplants, prevention of acute GVHD using immunosuppressants is necessary to decrease the incidence of GVHD and improve transplant outcomes. Specifically, tacrolimus, a calcineurin inhibitor, is commonly utilized for GVHD prophylaxis (Jacobson et al., 1998; Ram et al., 2009).

With a notably narrow therapeutic window and wide intra- and interindividual pharmacokinetic (PK) variability, initial weight-based dosing along with routine therapeutic drug monitoring of tacrolimus are employed to optimize its safety and efficacy. Both supratherapeutic and subtherapeutic tacrolimus concentrations can result in poor outcomes. Elevated steady-state trough concentrations of tacrolimus are associated with increased risk of adverse effects, including nephrotoxicity, hepatotoxicity, electrolyte abnormalities, and neurotoxicity, while subtherapeutic levels may increase the risk of developing GVHD and graft rejection (Kernan et al., 1986; Kernan et al., 1987; Butts et al., 2016; Andrews et al., 2018). Beyond intrinsically high PK variability, tacrolimus is primarily metabolized hepatically by cytochrome p450 3A4 and 3A5 (CYP3A4/5) and is, therefore, susceptible to many drug-drug interactions. Azole antifungals, used frequently post-HCT to prevent and treat fungal infections, are notorious inhibitors of CYP3A4/5, complicating the goal of reaching a steady, therapeutic concentration of tacrolimus. The clinical experience of the UCSF pediatric HCT center has found that current standard practice commonly results in high initial therapeutic trough levels of tacrolimus in its pediatric and young adult patients.

Tacrolimus PK variability is particularly important to consider in the pediatric population given the differences in absorption, distribution, metabolism and excretion among children of various sizes and at different stages of development (Kearns et al., 2003). Given the known variability in PK of tacrolimus in pediatric patients, several recently published studies have described pediatric-specific population pharmacokinetic (PopPK) models along with their associated dosing guides (Wallin et al., 2009; Andrews et al., 2018; Wang et al., 2020; Zhou et al., 2021). Zhou et al. developed a model within a subpopulation of children undergoing HCT for β-thalassemia (Zhou et al., 2021). Wang et al. and Wallin et al. described similar models where a one-compartment model best described the dataset and allometrically scaled weight improved approximations of clearance (Cl) and volume of distribution (Vd) (Wallin et al., 2009; Wang et al., 2020). Notably, Wang et al. solely examined patients receiving oral tacrolimus and Wallin et al. included a mix of IV and oral tacrolimus patients. While these models were developed in pediatric patients undergoing allogeneic HCT, given the small sample sizes and homogeneous populations of these studies, such approaches are unlikely to be generalizable to a broader population of children and young adults receiving HCT.

The objectives of the current study were to 1) develop a PopPK model for tacrolimus IV continuous infusion in the pediatric and young adult HCT population at UCSF Benioff Children’s Hospital and 2) implement model-informed dosing of tacrolimus utilizing the PopPK model in a clinically available Bayesian forecasting tool.

## Materials and Methods

### Patients and Data Collection

A retrospective chart review was conducted of all pediatric and young adult patients age <25 years treated at UCSF Benioff Children’s Hospital who received tacrolimus for GVHD prophylaxis during allogeneic HCT from February 2016 to July 2020. Patient characteristics were collected at the time of transplant and included age, gender, ethnicity, total body weight, and height. Transplant-specific characteristics included diagnosis, conditioning regimen, stem cell source, and degree of HLA mismatch. Though found significant in one published model, ursodeoxycholic acid was not evaluated in our model given little variability in its administration, as it is standard practice within the HCT center for nearly all patients to receive this medication (Wang et al., 2020). Transplant outcomes included presence of aGVHD or chronic GVHD (cGVHD), and survival at time of data collection. Tacrolimus-specific information included steady-state trough plasma concentrations for a minimum of 14 days of tacrolimus therapy (if available), time of tacrolimus therapy for which the plasma concentrations corresponded to, dosing rate of tacrolimus, and whether there was concomitant administration of voriconazole or posaconazole during each plasma concentration. In the context of this study, voriconazole or posaconazole were prescribed for prophylaxis or treatment of fungal infections.

### Tacrolimus Administration

The recommended starting dosing rate of tacrolimus for GVHD prophylaxis during HCT was 1.25 mcg/kg/h continuous IV infusion with a goal therapeutic range of 7–10 ng/ml. Steady-state tacrolimus plasma trough concentrations were typically measured every 24–48 h after initiation and every 24 h after a dosing change until the patient was consistently within the therapeutic range. The exact time of concentration measurements was not recorded and, therefore, it was assumed that each concentration was drawn the ideal 15 min before the next continuous infusion was started. Additionally, it was assumed that continuous infusions were given over exactly 24 h.

### Population Pharmacokinetic Modeling

The software utilized to build the PopPK model included NONMEM version v7.4 (ICON), PsN v4.8.1, and PiranaJS (beta version). Goodness of fit plots for model evaluation were created in R (v3.6.2) using the ggplot2 and vpc packages. The PopPK model building process included identifying structural and random effects models that best fit the data and then identifying which patient-specific covariates (if any) further improved model fit. Structural model exploration included evaluating fit of data to 1-compartment, 2-compartment, non-linear, and linear models. Both inter-individual and intra-individual variability was evaluated on all parameters and included if it could be estimated from the data and significantly improved fit. Additive, proportional, and combined residual error models were evaluated. Covariates that were available for evaluation were: weight, height, sex, age, co-treatment with CYP3A4/5 inhibitor (voriconazole and/or posaconazole), donor relation, aGVHD, cGVHD, and diagnosis. Weight was implemented allometrically. After implementation of weight, the other covariates were implemented and evaluated consecutively. Covariates were added using a stepwise approach with initial inclusion if covariate account was attributed to significant model fit improvement as quantified by a difference in OFV of -3.58 (*p* < 0.05). After initial covariate inclusion, each covariate was sequentially removed from the model and if any removal resulted in an improvement of fit associated with an alpha = 0.01, it was left out of the final model.

## Results

### Patient Characteristics

Data from 111 patients were collected and analyzed. The patients included in the dataset were found to be broadly reflective of the population treated at the HCT center, with median age of 7.3 years (range 0.5–25 years) and 66% of patients identified as non-Caucasian. The majority of patients were male (61%). Patients were treated for a variety of malignant and non-malignant diagnoses (Table 1). aGVHD was observed in 19 (17.1%) of patients and cGVHD was observed in 16 (14.4%) patients.

**TABLE 1:** Patient demographics and outcomes included in the PopPK tacrolimus continuous, IV infusion model.

Number of patients	111
Females sex number – n (%)	43 (39)
Age (years) – median (range)	7.3 (0.5 - 25)
Actual body weight (kg) – median (range)	23.9 (5.5 - 155.5)
Ancestry - n (%)
African American	6 (5.4)
American Indian or Alaskan Native	2 (1.8)
Asian/Caucasian/Hispanic	4 (3.6)
Asian	18 (16.2)
Caucasian/Non-Hispanic	38 (34.2)
Caucasian/Hispanic or Latino	38 (34.2)
Multi-ancestry	2 (1.8)
Other/Declined	3 (2.7)
Outcomes - n (%)
aGVHD	19 (17.1)
cGVHD	16 (14.4)
Deceased	21 (18.9)
Diagnosis - n (%)
Diagnoses Malignancies	65 (58.6)
Acute lymphoblastic leukemia	34 (30.6)
Acute myeloid leukemia	15 (13.5)
Juvenile myelomonocytic leukemia	8 (7.2)
Chronic myeloid leukemia	2 (1.8)
Myelodysplastic syndrome	3 (2.7)
Lymphoma	2 (1.8)
Natural Killer Cell Leukemia	1 (0.9)
Non-malignancies	46 (41.4)
Primary Immunodeficiencies	19 (17.1)
Aplastic Anemia/Bone Marrow Failure Syndromes	16 (14.4)
Inborn Errors of Metabolism	7 (6.3)
Hemoglobinopathies	4 (3.6)
Conditioning Regimen – n (%)
Busulfan/Fludarabine/Clofarabine	38 (34.3)
Busulfan/Fludarabine	14 (12.6)
Cyclophosphamide/Fludarabine	14 (12.6)
Cyclophosphamide/Total Body Irradiation	11 (9.9)
Melphalan/Fludarabine	10 (9.0)
Other	24 (21.6)
Serotherapy - n (%)
Antithymocyte globulin, rabbit	64 (57.7)
alemtuzumab	42 (37.8)
None	5 (4.5)
Donor Source - n (%)
Bone Marrow	55 (49.5)
Peripheral Blood Stem Cells*	49 (44.1)
Umbilical Cord Blood	7 (6.3)
Degree of HLA mismatch - n (%)
Fully Matched	62 (55.9)
1 Degree Mismatched	31 (27.9)
≥ 2 Degree Mismatched	18 (16.2)

*1 patient in the peripheral blood stem cell group also received bone marrow cells from the same donor.

**FIGURE 1:**
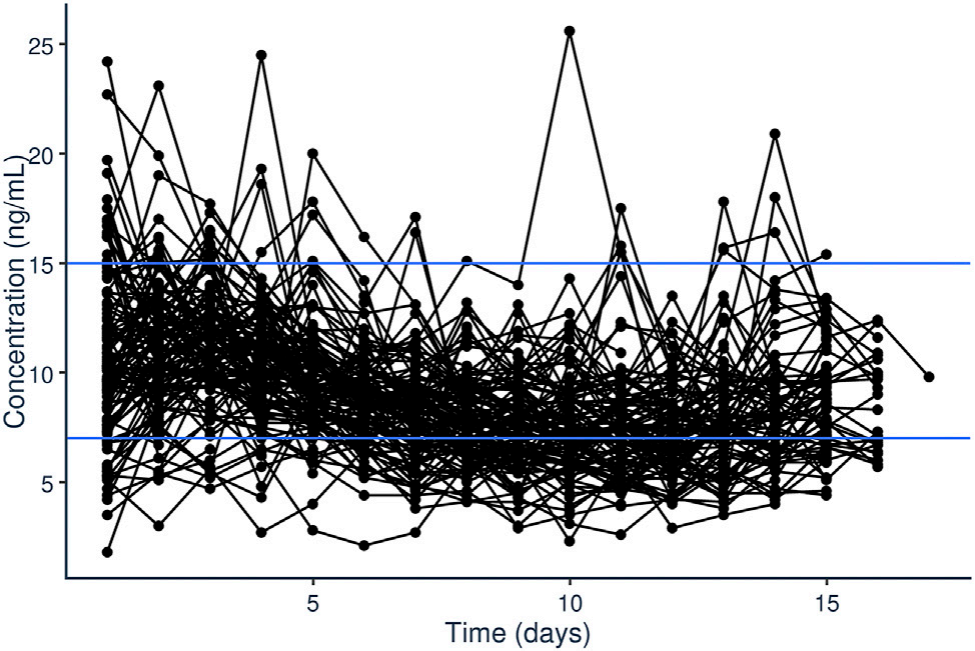
Steady-state trough plasma concentrations of tacrolimus (ng/ml) over time (days) after initiation of tacrolimus IV continuous infusion in pediatric patients undergoing HCT.

A total of 1,648 tacrolimus plasma steady-state trough concentrations were included in the PopPK modeling process. Trough levels were performed for a median of 14 days after starting tacrolimus continuous IV infusion, though not every patient had levels consistently drawn each day for the first 14 days of treatment. The median tacrolimus dosing rate was 1.25 mcg/kg/h with a range of 0.45 mcg/kg/h to 1.25 mg/kg/h. A total of 929 (56.4%) of the 1,648 samples within the first 14 days were outside of the goal of 7–10 ng/ml (Figure 1). For 193 (11.7%) of the 1,648 samples, voriconazole or posaconazole was administered concomitantly. The median first steady-state trough concentration was 10.2 ng/ml (range 1.8–24.2 ng/ml).

**FIGURE 2:**
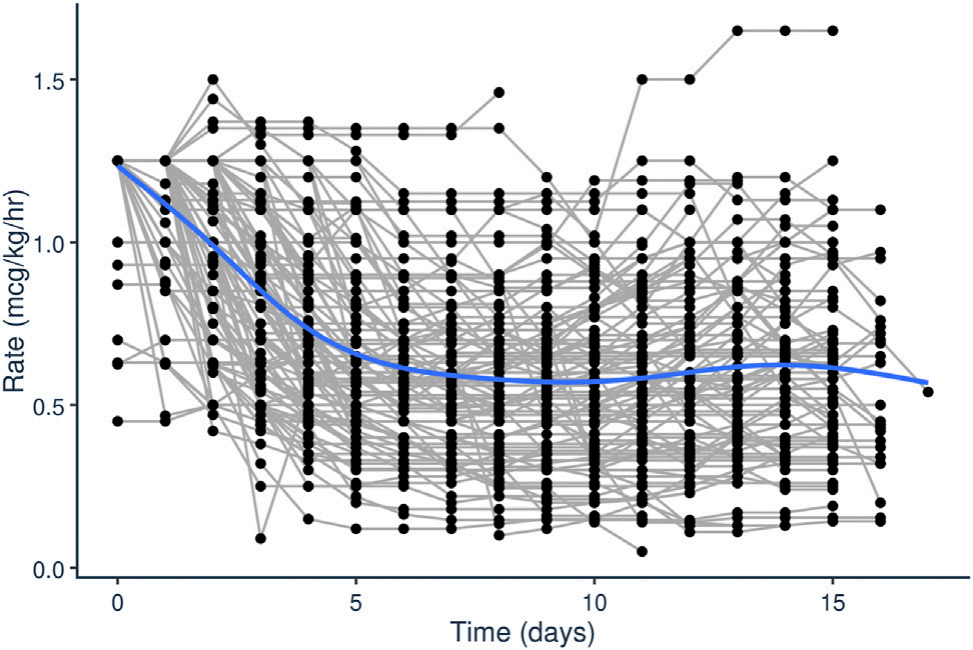
Tacrolimus IV infusion rage (mcg/kg/h) over time (days) after initiation of tacrolimus IV continuous infusion in pediatric patients undergoing HCT.

Initial dataset investigation informed the PopPK model building process. It became clear that, on average, the fixed initial dosing rate of 1.25 mcg/kg/h generally resulted in initial supratherapeutic tacrolimus trough levels as evident by the median initial trough concentration of 10.2 ng/ml (Figure 1). Additionally, looking at the average dosing rate over time showed a distinct decrease in dosing rate over the first few days of treatment which eventually plateaued (Figure 2). Specific patient characteristics were then evaluated to probe for potential covariates driving the variation which led to patients being over-dosed at the fixed total body weight institutional practice.

### Population Pharmacokinetic Model

A two-compartment model with fixed Q and V2 showed improved model fit (*p* < 0.001) over a one-compartment model. Intercompartmental clearance (Q) and peripheral compartment volume (V2) were fixed to Cl and Vd by a fixed multiplication factor (Fact). Fact was determined based on the average of several published population PK studies, and it was assumed to be the same for both Q and V2 (Xue et al., 2011; Kassir et al., 2014; Moes et al., 2016; Andrews et al., 2018; Andrews et al., 2020). Non-linear clearance was found to lead to significantly better fit, although the improvement in fit was not apparent from any of the goodness of fit plots (plots not included in this report). For the non-linear model iterations, Michaelis constant (Km) was estimated around 10 ng/ml; however, we did not include the non-linear component in the final model. Of note, inclusion of a time-dependent effect on CL did not improve model fit for the 2-compartment model, and only slightly for the 1-compartment model.

In terms of accounting for random effects, between-patient variability was estimated on both CL and V, and improved fit significantly for both parameters and so was included in the final model. Inter-occasion variability was estimated on both CL and V, but only led to significant improvement in fit for CL (and due to fixed implementation of Q, also on Q). A residual error with both proportional and additive components could not be identified from the data. Therefore, only the proportional component was retained, which was estimated to be 17.9%.

Patient-specific covariates were identified which improved model fit upon inclusion. Actual body weight, implemented using allometric principles on all PK parameters, was a significant covariate in the model. Estimation of the allometric parameters only led to moderate improvements in fit and were estimated at close to the theoretic exponents (0.73 vs 0.75 for CL, and 0.83 vs 1.0 for V), so they were fixed at their theoretic values. Use of allometry based on fat-free-mass instead of weight did not improve fit. Co-treatment with a CYP-inhibitor significantly reduced the clearance of tacrolimus (*p* = 0.001, estimate 20%, on clearance 95% CI: 10–33%). No other covariates were found to be predictive factors on either clearance or volume parameters.

As no sensible groupings were identified that would have resulted in relevant group sizes to allow estimation of a covariate effect for diagnosis, it was not attempted to estimate the effect of diagnosis on PK parameters. Likewise, ancestry was evaluated but we did not have large enough group sizes to estimate a covariate effect. None of the transplant-specific characteristics or outcomes (donor, antigen mismatch, aGvHD, cGvHD, alive status) showed significance. Additionally, survival status was not a significant outcome as most recipients were alive (81%) at the time of data collection.

### Model Validation

**FIGURE 3:**
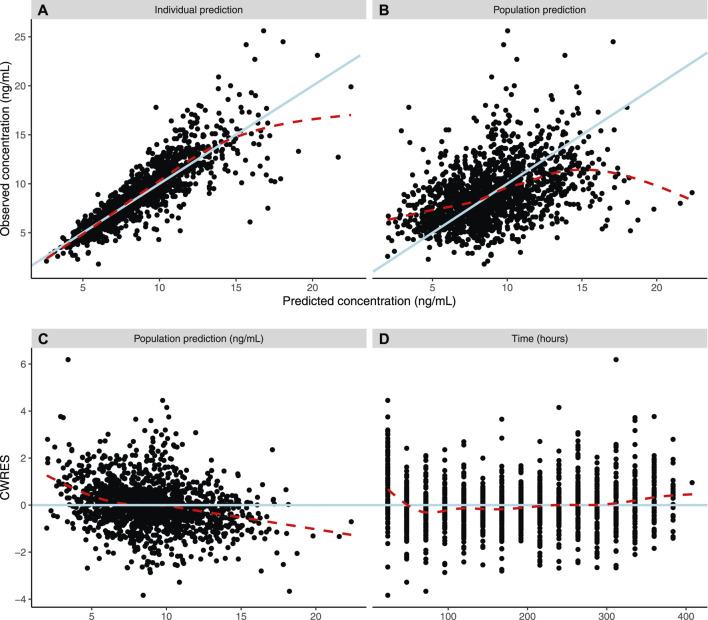
Population pharmacokinetic model diagnostic plots. Dashed lines show a weighted average, while solid lines indicate line of unity (3AB) or line of zero residual (3CD). **(A)** Actual individual tacrolimus steady-state plasma concentrations (ng/ml) versus model-guided individual prediction of tacrolimus steady-state plasma concentrations (ng/ml) taking into account random effects and individual factors. **(B)** Actual individual tacrolimus steady-state plasma concentrations (IPRED) (ng/ml) versus model-guided population predictions of tacrolimus steady-state plasma concentrations (PRED) (ng/ml). **(C)** Conditional weighted residuals (CWRES) (%) over PRED (ng/ml) of tacrolimus. **(D)** CWRES (%) over time (hours).

**FIGURE 4:**
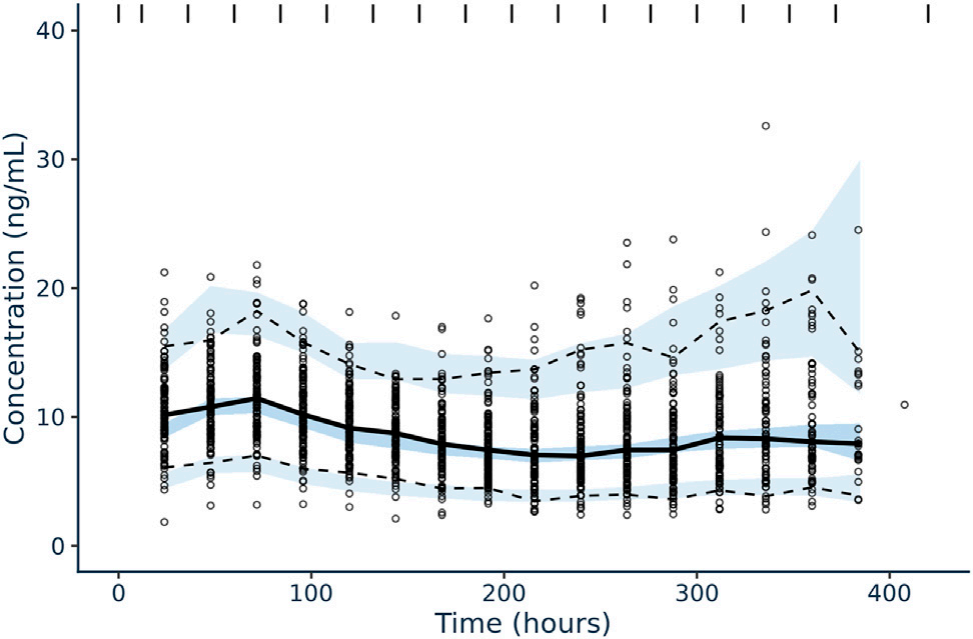
Confidence interval visual predictive check for tacrolimus IV, continuous infusion population pharmacokinetic model in pediatric patients undergoing HCT. Solid black line corresponds to the median plasma concentrations (ng/ml) of tacrolimus over time (hour) and the dotted black lines correspond to the 5% (lower) and 95% plasma concentration (ng/ml) intervals of concentrations included in the dataset utilized for model building. The blue shaded areas correspond to the model-guided predictions of 5%, median, and 95% plasma concentrations (ng/ml) intervals over time.

Diagnostic plots and a visual predictive check were utilized to validate the model (Figures 3, 4). Overall, model predictions only showed moderate bias, with minor under-prediction at lower concentrations and minor over-prediction at higher predicted concentrations. The VPC plot showed adequate prediction of the median and 5th/95th percentiles over time, with only a slight under-prediction of the median for the concentration at day 1 of treatment (Figures 3, 4). The final model was therefore defined as:

$$CL_{i}=\theta_{CL}\cdot {\left(WT_{i}/70\right)}^{0.75}\cdot \theta_{inh}^{INH}\cdot \exp\left(\eta_{CL_{i}}+\kappa_{j}\right)$$

$$V_{1,i}=\theta_{V_{1}}\cdot {\left(WT_{i}/70\right)}^{1}\cdot \exp\left(\eta_{V_{1,i}}\right)$$

$$Q_{i,j}=CL_{i,j}*Fact$$

$$V_{2,i}=V_{1,i}*Fact$$

The final parameter estimates are reported in Table 2.

**TABLE 2:** Population pharmacokinetic parameter estimates of final model.

Parameter	Value	RSE	Unit	IIV	IOV
*θ*_CL_	4.2	2.95%	L/h	26.1%	28.7%
*θ*_V_	61.9	5.98%	L	-	-
*θ*_INH_	0.8	6.97%		-	-
Fact	2.0	fixed		-	-

## Discussion

While the standard practice at most transplant centers to therapeutic drug monitoring for tacrolimus is essentially a “guess and check” approach using standard weight-based dosing, our center has observed that initial tacrolimus levels are frequently supratherapeutic. This clinical finding led us to hypothesize that a model-informed, precision-based approach to dosing of continuous, IV tacrolimus could enable us to reach our goal therapeutic range more quickly and maintain these levels consistently throughout treatment. Therefore, we developed this PopPK model with the intention of optimizing tacrolimus dosing to improve its safety and effectiveness in clinical practice.

The developed PopPK model has several advantages for our patient population as compared to the current models available in the literature. First, it was developed in a pediatric and young adult population for which it is intended to be used, i.e., continuous infusion for patients undergoing HCT. Furthermore, it was developed from a broad population of patients including diverse age range, ethnicities, and indications for HCT, supporting the generalizability of the model for the population undergoing HCT at the UCSF Benioff Children’s Hospital and other large transplant centers. Additionally, the inclusion of voriconazole and posaconazole in covariate analysis was novel from previously published PopPK models of tacrolimus in this population and provides further utility for this approach. Moreover, the PopPK’s clinical utility in conjunction with its build into a Bayesian forecasting platform enables smooth translation into improving patient outcomes. Further fine tuning and improvement of the model within the Bayesian forecasting platform is readily attainable, as we have previously demonstrated for other drugs in HCT (Shukla et al., 2020).

Although only concentrations from continuous dosing of tacrolimus were available, we selected a 2-compartment model, as we felt there was significant scientific justification for this decision given that prior publications of tacrolimus PopPK models have demonstrated two-compartment kinetics. Within the pediatric renal transplant setting, Andrews et al. developed a PopPK model of tacrolimus which fit a two-compartment structural model with patient-specific covariates including allometrically scaled weight, CYP3A5 isoform status, hematocrit, estimated glomerular filtration rate, and donor living status factored into tacrolimus clearance (Andrews et al., 2018). While insightful, there are some key disease-state differences that make extrapolation of this model to the HCT setting difficult, including the reliance on IV tacrolimus for extended periods post-HCT, high potential for liver injury secondary to receiving high-dose chemotherapy and radiation in HCT, and difference in intended purpose (preventing both graft rejection and GVHD).

Technically, if the model is only to be used for prediction of steady state levels, which is indeed its intended use, it would not matter if a 1- or 2-compartment model would be selected. However, the more physiologically valid 2-compartment model is expected to be more predictive in clinical situations that deviate from the anticipated use, such as when the tacrolimus continuous infusion would be stopped for some time or given intermittently. For the non-linear PK iterations of the model building process, the Km was estimated to be in the middle of the clinically observed concentration range for continuous IV, lending credibility to the non-linear model. However, as far as we know, no other population PK analyses on tacrolimus have reported non-linear PK in the clinically observed concentration range for common dosage regimens. Given that the current analysis included only sparse data (troughs), obtained only from continuous IV administration, and that the dataset was of relatively limited size (*n* = 111), we were hesitant to conclude that tacrolimus continuous IV, given at the dosages in this cohort, leads to non-linear PK. Therefore, we did not include the non-linear component in the final model, but we report the Km here for future reference.

While not included in the final two-compartment model, the time effect on clearance, which improved fit of the one-compartment model, is interesting to note. This phenomenon matches clinical experience in which hepatotoxicity secondary to the conditioning regimen progresses on average over the first 2 weeks after transplant and during the time of tacrolimus data collection in our dataset. Since tacrolimus elimination is dependent on the cytochrome P450 system within the liver, hepatocellular toxicity due to high dose chemotherapy prior to HCT resulting in impaired metabolic activity is a logical explanation for such a time-dependent decrease in tacrolimus clearance after HCT. Further studies elucidating the relationship between hepatoxicity secondary to conditioning regimen and tacrolimus clearance are warranted.

In current published PopPK models within the pediatric HCT population, there is some diversity in evaluated and included covariates. Given tacrolimus’ hepatic metabolism and the possibility of hepatotoxicity secondary to conditioning in this population, inclusion of liver function tests as covariates and status of veno-occlusive disease/sinusoidal obstruction syndrome as a clinical outcome within the Bayesian forecasting platform may improve the model further. Inclusion of greater numbers of the representative ancestry groups will help to elucidate any potential effect on tacrolimus PK. Likewise, the inclusion of CYP3A4/5 genotyping into the model will likely improve the model significantly given its well documented relationship with tacrolimus PK and pharmacodynamics within the population (Birdwell et al., 2015). While it was not standard clinical practice at the time of data collection for this study, as recent studies support the use of CYP3A5 genotyping in patients undergoing allogeneic HCT (Zhu et al., 2020; Yoshikawa et al., 2021), our group plans to obtain this information for future patients as part of our standard clinical practice, which will further help to guide tacrolimus dosing within the PopPK model. We plan to prospectively evaluate the model-informed dosing of tacrolimus IV continuous infusion in the pediatric HCT population at UCSF Benioff Children’s Hospital as well as continue to improve the model over time as more patient data is collected.

## Conclusion

Prevention of aGVHD after allogeneic HCT is clinically important as this complication is a leading cause of post-HCT morbidity and mortality. Development of a PopPK model of tacrolimus elucidates factors driving variability within this population and accounts for patient-specific covariates to guide initial dosing rate. The described 2-compartment model accounts for relevant patient covariates including allometrically scaled weight and presence of a significant CYP3A4/5 inhibitor. Additionally, implementation of such a model in a Bayesian dosing platform translates this research directly into a clinically impactful application, that has the potential to improve outcomes for pediatric and young adult patients.

## Data Availability

The raw data supporting the conclusions of this article will be made available by the authors, without undue reservation.

## Abbreviations

Cl, Clearance; CYP, Cytochrome P450; DDI, Drug-drug interaction; GVHD, Graft-versus-host disease; HCT, Hematopoietic stem cell transplant; Km, Michaelis constant; PK, Pharmacokinetic; PopPK, Population pharmacokinetic; Vd, Volume of distribution.
